# Hepatic steatosis induced in C57BL/6 mice by a non-ß oxidizable fatty acid analogue is associated with reduced plasma kynurenine metabolites and a modified hepatic NAD^+^/NADH ratio

**DOI:** 10.1186/s12944-020-01271-1

**Published:** 2020-05-14

**Authors:** Rolf K. Berge, Daniel Cacabelos, Rosa Señarís, Jan Erik Nordrehaug, Ottar Nygård, Jon Skorve, Bodil Bjørndal

**Affiliations:** 1 Department of Clinical Science, University of Bergen, Bergen, Norway; 2 Department of Heart Disease, Haukeland University Hospital, Bergen, Norway; 3 Department of Physiology, CIMUS, University of Santiago de Compostela-Instituto de Investigación Sanitaria, Santiago de Compostela, Spain; 4 Department of Heart Disease, Stavanger University Hospital, Stavanger, Norway; 5 KG Jebsen Centre for Diabetes Research, University of Bergen, Bergen, Norway

**Keywords:** Non-alcoholic fatty liver, Kynurenine metabolites, Hepatic NAD metabolism, Mouse model

## Abstract

**Background:**

Non-alcoholic fatty liver disease is often associated with obesity, insulin resistance, dyslipidemia, and the metabolic syndrome in addition to mitochondrial dysfunction and nicotinamide adenine dinucleotide (NAD^+^) deficiency. The aim of this study was to investigate how inhibition of mitochondrial fatty acid oxidation using the compound tetradecylthiopropionic acid (TTP) would affect hepatic triacylglycerol level and plasma levels of kynurenine (Kyn) metabolites and nicotinamide.

**Methods:**

12 C57BL/6 mice were fed a control diet, or an intervention diet supplemented with 0.9% (w/w) tetradecylthiopropionic acid for 14 days. Blood and liver samples were collected, enzyme activities and gene expression were analyzed in liver, in addition to fatty acid composition. Metabolites in the tryptophan/kynurenine pathway and total antioxidant status were measured in plasma.

**Results:**

Dietary treatment with tetradecylthiopropionic acid for 2 weeks induced fatty liver accompanied by decreased mitochondrial fatty acid oxidation. The liver content of the oxidized form of NAD^+^ was increased, as well as the ratio of NAD^+^/NADH, and these changes were associated by increased hepatic mRNA levels of NAD synthetase and nicotinamide mononucleotide adenyltransferase-3. The downstream metabolites of kynurenine were reduced in plasma whereas the plasma nicotinamide content was increased. Some effects on inflammation and oxidative stress was observed in the liver, while the plasma antioxidant capacity was increased. This was accompanied by a reduced plasma ratio of kynurenine/tryptophan. In addition, a significant decrease in the inflammation-related arachidonic fatty acid in liver was observed.

**Conclusion:**

Fatty liver induced by short-time treatment with tetradecylthiopropionic acid decreased the levels of kynurenine metabolites but increased the plasma levels of NAD^+^ and nicotinamide. These changes are most likely not associated with increased inflammation and oxidative stress. Most probably the increase of NAD^+^ and nicotinamide are generated through the Preiss Handler pathway and/or salvage pathway and not through the de novo pathway.

**The take home message** is that non-alcoholic fatty liver disease is associated with the metabolic syndrome in addition to mitochondrial dysfunction and nicotinamide adenine dinucleotide (NAD^+^) deficiency. Inducing fatty liver in mice by inhibition of fatty acid oxidation resulted in a concomitant change in kynurenine metabolites increasing the plasma levels of nicotinamides and the hepatic NAD^+^/NADH ratio, probably without affecting the de novo pathway of kynurenines.

## Background

**Fig. 1:**
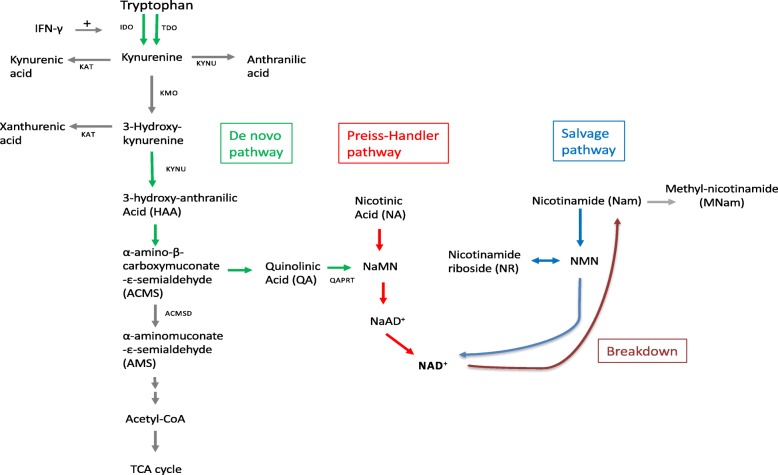
Schematic representation of tryptophan -and nicotinamide pathways. Abbreviations: NaAD, nicotinic acid adenine dinucleotide; NAD, nicotinamide adenine dinucleotide; NaMn, nicotinic acid mononucleotide; NMN, nicotinamide mononucleotide

The redox state is an important factor in regulating metabolic reactions, cellular signalling pathways and stress reaction systems. Mitochondria are the major source of reactive oxygen species and both the oxidized form of nicotinamide (NAD^+^) and the reduced form NADH are important cofactors for the redox state. De novo biosynthesis of NAD takes place through tryptophan (Trp) catabolism by the kynurenine (Kyn) pathway (Fig. 1). Mitochondrial fatty acid oxidation needs NAD^+^ as a cofactor, and interestingly, the enzymes responsible for the generation of Kyn metabolites are present in both mitochondria and/or cytosol [1, 2]. Therefore, alterations in the Trp-metabolism, such as changes in enzyme activity or gene expression as well as substrate availability may influence mitochondrial function or vice versa.

Kyn is synthesized by indoleamine 2.3-dioxygenase (IDO) from Trp which accounts for more than 90% of the Trp catabolism [3]. Trp can also be converted to Kyn by tryptophan 2.3-dioxygenase (TDO). Thus, the plasma Kyn/Trp ratio (KTR), which increases during inflammation [4], is influenced by the activities of both IDO and TDO. The cytokine interferon-γ (IFN-γ) stimulates the conversion of Kyn from Trp by up-regulating IDO [3]. IFN-γ also stimulates the formation of neopterin in macrophages [4, 5]. After conversion of Trp to Kyn, Kyn is metabolized further to 3-hydroxykynurenine (HK) by the enzyme kynurenine monooxygenase (KMO). Kyn may also be converted to kynurenic acid (KA) by kynurenine aminotransferase (KAT), or to anthranilic acid (AA) by kynureninase (KYNU). Similarly, KAT converts 3-hydroxykynurenine (HK) to xanthurenic acid (XA) or to 3-hydroxyanthranilic acid (HAA) by KYNU (Fig. 1). The plasma levels of Kyn, HK, HAA, KA, or the Kyn/Trp ratio have been reported to be associated with chronic and acute inflammation [6, 7], and predict risk of early death and long-term outcome in patients resuscitated from cardiac arrest [8].

HAA is used for further biosynthesis of NAD+. First HAA is converted to α-amino-β-carboxy muconate-ε-semialdehyde (ACMS). Then ACMS is further converted to quinolinic acid (QA), nicotinic acid mononucleotide (NaMn) and NAD+. Nam is a product of NAD degradation and can be recycled into NAD biosynthesis. Nam can also be degraded to N1-methylnicotinamide (mNam).

It is important to note that aminocarboxymuconate-semialdehyde decarboxylase (ACMSD) degrades ACMS into α-aminomuconate-ε-semialdehyde, a precursor for the glutarate pathway, thereby diverting Trp catabolites from NAD synthesis through QA. In addition, nicotinic acid obtained from the diet will be a precursor for NAD synthesis through its metabolite NaMN, also called the Preiss-Handler pathway (Fig. 1).

Non-alcoholic fatty liver disease (NAFLD) is defined as accumulation of lipids in the liver. The prevalence of NAFLD is increasing worldwide [9], as it is often associated with obesity, insulin resistance, dyslipidemia, the metabolic syndrome, and cardiovascular risk [10]. NAFLD patients exhibit signs of liver inflammation [11] and dysregulated mitochondrial metabolism is central in hepatic steatosis. It is also reported that Trp alone and its metabolites are involved in the development of fatty liver when injected in rats [12], but recently, Ritze and collaborators reported that oral Trp supplementation reduces diet-induced NAFLD in mice [13].

Tetradecylthiopropionic acid (TTP) is a saturated fatty acid with a sulphur atom in the 4-position from the carboxylic end. This implies that the fatty acid derivative can undergo one cycle of mitochondrial β-oxidation. TTP is a weak peroxisome proliferator but inhibits the mitochondrial β-oxidation [14–16]. Recently, we suggested that TTP-induced hepatic steatosis occurred when the amount of imported and synthesized lipids exceeded the export or catabolism in hepatocytes [17]. It has been assumed that limited mitochondrial fatty acid oxidation is a common feature in NAFLD. Little is known about the influence of TTP-induced fatty liver on the pattern of metabolites along Trp catabolic pathway and Trp-Nam pathway.

Unlike most other tissues the liver uses a substantial amount of dietary Trp to synthesize NAD [18]. Given that mitochondria are affected in NAFLD, with a decreased fatty acid oxidation and a modified NAD metabolism, the aim of the study was to investigate the relationship between mitochondrial fatty acid β-oxidation, the kynurenine pathway and the synthesis of NAD in vivo in mice.

## Materials and methods

### Animals

12 weeks old C57BL/6 (Taconic, Denmark) male mice were housed in Makrolon III cages, 2–3 animals per cage, in an open system. They were kept under standard laboratory conditions with temperature 22 ± 1 °C, dark/light cycles of 12/12 h, relative humidity 55 ± 5% and 20 air changes per h. The animal study was conducted according to the Guidelines for the Care and Use of Experimental Animals, and the Norwegian State Board of Biological Experiments with Living Animals approved the protocol.

Animals were divided at random into two groups of 6 mice, and after 7 days of acclimatization control groups were fed a diet with 7% fat (5% lard and 2% soy oil) while the intervention diet was supplemented with 0.9% TTP [17]. All groups had free access to tap water and food during the 14 days experiment. On day 14, all mice were anesthetized by inhalation of 2–4% Isofluorane (Forane from Abbot Laboratories Ltd. Illinois. USA) and thoracotomy, cardiac puncture, and exsanguination was performed. Plasma and liver samples were stored at − 80 °C.

### Hepatic enzyme activities

At sacrifice, livers were removed. Chilled on ice. Weighed and snap-frozen in liquid nitrogen. 100 mg liver from each rat was homogenized in 1 mL ice-cold sucrose medium (0.25 M sucrose. 10 mM HEPES. and 1 mM Na_4_EDTA, adjusted to a pH of 7.4 with KOH) giving 10% (w/v). The homogenates were centrifuged at 600 g-force for 10 min at 4 °C and the post-nuclear fraction was removed and used for further analysis. The assay for in vitro fatty acid oxidation, using palmtoyl-CoA as substrate, was performed according to [17, 19]. In vitro oxidation of palmitoyl-CoA was calculated in nmol/min/g liver. The activity of fatty acyl-CoA oxidase (ACOX) was measured in post-nuclear fractions, as described by [20].

### Analysis of metabolites and fatty acid composition in plasma and liver

Liver lipids were extracted according to Bligh and Dyer [21], evaporated under nitrogen, and redissolved in isopropanol before analysis. Lipids from liver extracts were then measured enzymatically on a Hitachi 917 system (Roche Diagnostics GmbH, Mannheim. Germany) using the triacylglycerol (GPO-PAP) kit. The liver fatty acids including arachidonic acid (C20:4n-6) content was analyzed as described previously [22]. The anti-inflammatory fatty acid index was calculated according to the formula: ((20:5n-3 + 20.3n-6 + 22:6n- 3/20:4n-6)) × 100. The atherogenicity index (AI) was determined according to Ulbricht and Southgate [23] as follows: AI = (C12:0 + 4*C14:0 + C16:0)/ (ΣMUFA + Σ(n-6 PUFA) + Σ(n-3 PUFA)), where ΣMUFA, Σ(n-6 PUFA), and Σ(n-3 PUFA) are the sum of MUFA (monounsaturated fatty acids), n-6 and n-3 PUFA (polyunsaturated fatty acids) in wt% of total fatty acids, respectively.

Neopterin, Trp and six kynurenines (Kyn, AA, KA, HK, HAA, and XA) were measured in plasma samples using a high-throughput liquid chromatography tandem mass spectrometry assay according to Midthun et al. [24, 25]. KTR was calculated by dividing the plasma concentration of Kyn by the concentration of Trp and subsequently multiplying by 1000.

Total antioxidant capacity of plasma was measured using the total antioxidant capacity kit (Abcam, Cambridge, UK) according to the manufacturer’s instructions. The protein mask was not used, enabling the analysis of both small molecule antioxidants and proteins ability to reduce Cu2+ to Cu+. In brief, EDTA-plasma was allowed to reduce Cu2+ for 1.5 h at room temperature on an orbital shaker. The absorbance was measured at 570 nm using a plate reader. Results were expressed as trolox equivalents according to a trolox standard curve.

NAD^+^/NADH concentrations and ratios were measured using the NAD/NADH Quantification Colorimetric Kit (ABIN411692, Antibodies-online.com) according to the manufacturer’s instructions. Snap cryo-frozen liver samples stored at − 80 were used to make fresh tissue lysates; 20 mg of liver was homogenized with 400 μL of NADH/NAD extraction buffer. To remove enzymes that could consume NADH rapidly the samples were filtered through 10 Kd molecular weight cut off filters before performing the assay. To detect NADH, NAD was decomposed by heating extracted samples to 60 °C for 30 min in a heating block. Concentrations were determined from a NADH standard curve.

### Histochemistry analysis

Cryo-sections from frozen livers were generated using a 1720 Cryostat (Leica Microsystems, Wetzlar, Germany). Section were fixed in 4% buffered formalin for 10 min. Rinsed 3x in dH2O, before staining in 0.7% (w/v) Oil Red O (Sigma) in propylene glycol for 10 min, rinsed 3x dH2O, and stained with hematoxylin (Thermo Fisher Scientific. Waltham. MA. USA) for 2 min. Finally, sections were rinsed 3x dH2O and mounted with ImmuMount (Thermo Fisher Scientific). Images were captured using an Olympus BX51 light microscope at 40x magnification with an Olympus DP25 digital color camera (Olympus Corporation, Tokyo, Japan). Three images were captured from each animal by a blinded investigator.

### Gene expression analysis

Total cellular RNA was purified from frozen liver samples, and cDNA was produced as previously described [26]. Real-time PCR was performed with Sarstedt 384 well multiply-PCR Plates (Sarstedt Inc., Newton, NC, USA) on the following genes, using probes and primers from Applied Biosystems: Aminoadipate aminotransferase/kynurenine aminotransferase II (*Aadat/KatII/Kyat2*, Mm00496169_m1), Aminocarboxymuconate semialdehyde decarboxylase (*Acmsd*, Mm01291680_m1), Adhesion G protein-coupled receptor E1 (*Adgre1 (F4/80)*, Mm00802529_m1), Catalase (*Cat*, Mm00437992_m1), Idoleamine 2,3-dioxygenase 1 (*Ido1*, Mm00492586_m1), Indoleamine 2,3-dioxygenase 2 (*Ido2*, Mm00524210_m1), Kynurenine 3-monooxygenase (*Kmo/Kat3mo*, Mm01321343_m1), Kynureninase (*Kynu*, Mm00551012_m1), NAD kinase *(Nadk*, Mm00446804_m1), NAD kinase 2, mitochondrial (*Nadk2*, Mm01297768_m1), NAD synthetase 1 (*Nadsyn1*, Mm00513448_m1), Nicotineamide phosphoribosyltransferase (*Nampt*, Mm00451938_m1), Nicotinamide nucleotide adenylyltransferase 1 (*Nmnat1*, Mm01257929_m1), Nicotinamide nucleotide adenylyltransferase 2 (*Nmnat2*, Mm00615393_m1), Nicotinamide Nucleotide Adenylyltransferase 3 (*Nmnat3,* Mm00513791_m1), Quinolinate phosphoribosyltransferase (*Qprt*, Mm00504998_g1), Tryptophan 2,3-dioxygenase (*Tdo2*, Mm00451266_m1). Three different reference genes were included: 18 *s* (Kit-FAM-TAMRA (Reference RT-CKFT-18 s)) from Eurogentec (Liège, Belgium), glyceraldehyde-3-phosphate dehydrogenase (*Gapdh*, Mm99999915_g1) from Applied Biosystems, and ribosomal protein, large, P0 (*Rplp0*, Gene ID 11837) from Thermo Fisher Scientific. The NormFinder software was used to evaluate the reference genes [27], and data normalized to *18 s* are presented.

### Statistical analysis

Data sets were analyzed using Prism Software (Graph-Pad Software, San Diego, CA) to determine statistical significance. The results are shown as means of 6 animals per group with their standard deviations. Normal distribution was determined by the Kolmogorov-Smirnov test (with Dallal-Wilkinson-Lilliefor *P* value). Either an unpaired t-test was performed to evaluate statistical differences between groups, or Mann Whitney test when values were not normally distributed. Correlation between variables was evaluated by the Pearson’s statistic, *P* -values < 0.05 were considered significant.

## Results

### TTP increases triacylglycerol (TAG) level and reduces mitochondrial fatty acid oxidation in liver without association with oxidative stress and inflammation

**Fig. 2:**
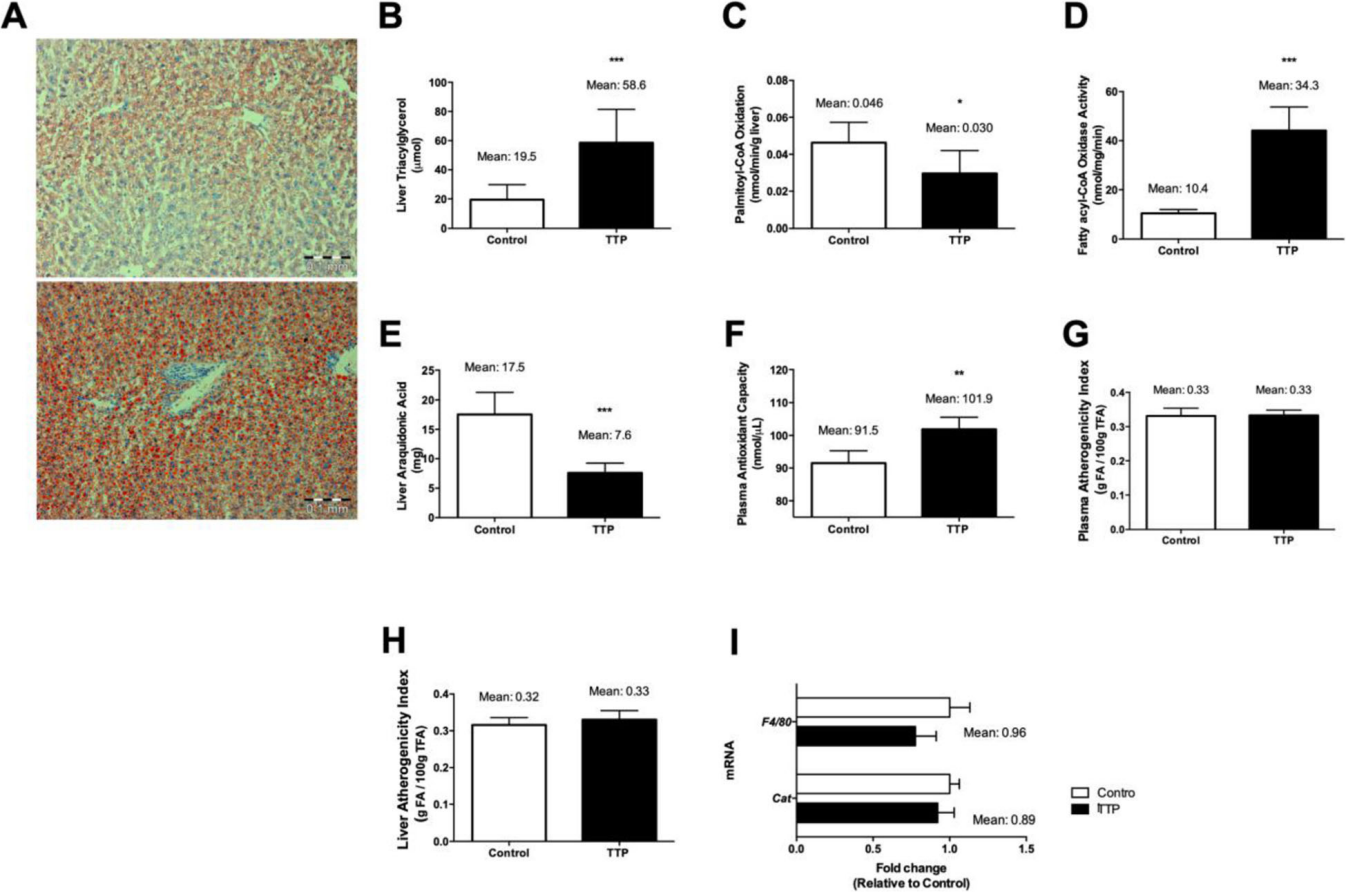
Fatty liver analysis, gene and metabolic indexes related to its oxidative status. **a** Representative histological images showing liver lipid droplet accumulation under experimental conditions. Control and TTP treated C57BL/6 male liver sections were frozen and stained with oil-red. **b** Total liver triacylglycerol accretion along dietary intervention. **c** In vitro palmitoyl-CoA oxidation analysis. **d** Fatty acyl-CoA oxidase activity was performed in liver post-nuclear fractions. **e** Total liver arachidonic acid accumulation in C57BL/6 males. **f** Plasma antioxidant capacity. **g** Plasma and **h** liver atherogenicity indexes were calculated from lipid profile. **i** Gene expression analysis in liver. Data presented are mean ± standard deviation (from six animals per group). Statistical significance between control and TTP was shown as: *P < 0.05; **P < 0.01; ***P < 0.001

The feed intake, feed efficiency, body weight, as well as liver weight were not affected by TTP administration in mice (data not shown), but oil-red-O staining indicated that hepatic steatosis was induced (Fig. 2a). This was accompanied by increased total hepatic triacylglycerol (TAG) content (Fig. 2b) and decreased in vitro hepatic fatty acid oxidation of palmitoyl-CoA (Fig. 2c). Interestingly, however, the peroxisomal fatty acyl-CoA oxidase (ACOX) activity was increased (Fig. 2d). Due to the ability of TTP to develop fatty liver it was of interest to investigate markers of inflammation and oxidative stress in plasma and liver. The total content of arachidonic acid C20:4n-6) in liver was decreased by about 40% compared to control Fig. 2e), whereas the total antioxidant capacity in plasma increased and the plasma and liver atherogenicity fatty acid index was unchanged (Fig. 2f, g and h, respectively). In agreement with previous findings, both liver and plasma anti-inflammatory fatty acid index increased [17]. The hepatic gene expression level of *F4/80*, a marker of macrophage infiltration, as well as catalase gene expression was not significantly increased by TTP treatment (Fig. 2i). Moreover, no significant increases in the gene expression levels of hepatic inflammatory mediators, including *Ifn-γ* and *Il-*6 mRNA, resulted after TTP administration (data not shown). Previous findings have shown that TTP administration reduced the hepatic gene expression of *Tnfα* but increased the mRNA level of superoxide dismutase 1 [17]. Altogether, these results suggest that hepatic steatosis induced by 2 weeks TTP-treatment is not associated with increased markers of inflammation and oxidative stress in liver or in the circulation.

### Plasma metabolites in the Trp-Kyn pathway and associated liver gene expression is influenced by TTP treatment

**Fig. 3:**
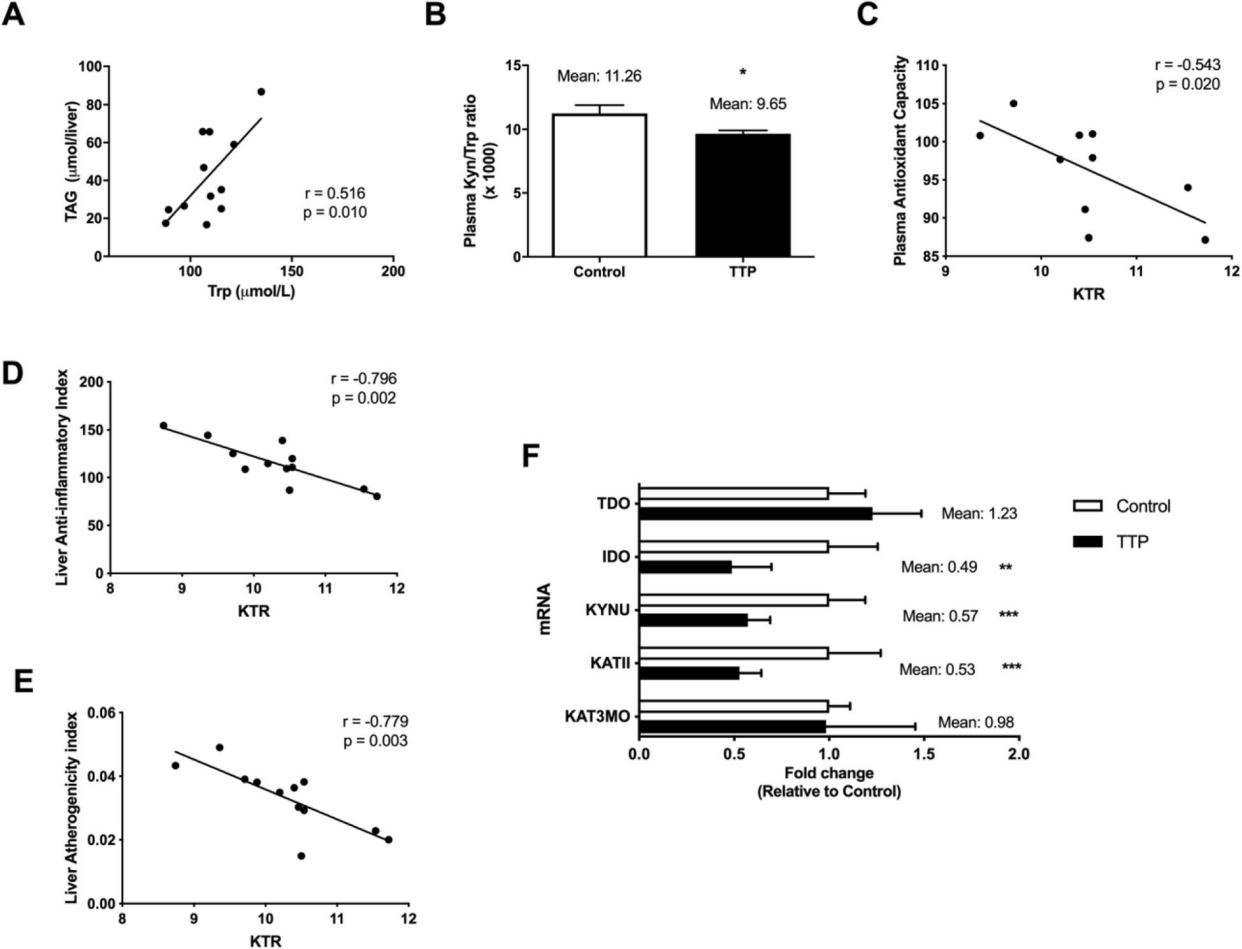
Plasma metabolites in the tryptophan (Trp)- kynurenine (Kyn) pathway, relationship with inflammation indexes, and liver gene expressions. **a** Correlation analysis between TAG and Trp concentration in plasma. **b** Plasma Kyn/Trp ratio × 1000 (KTR). Correlation analysis for plasma Kyn/Trp ratio and (**c**) plasma antioxidant capacity, (**d**) liver anti-inflammatory index, and (**e**) liver atherogenicity index. **f** mRNA expression analysis in liver. Data represented mean ± standard deviation. Statistical significance between control and TTP was shown as: * *P* < 0.05; ** *P* < 0.02; *** *P* < 0.001

Given that Trp-metabolites may be involved in the development of fatty liver and inflammation we investigated how TTP affected metabolites of the Trp-Kyn pathway in plasma and the gene expression of relevant enzymes in liver. The plasma concentrations of Trp and Kyn were increased under dietary TTP administration (Table 1) and a positive correlation between plasma Trp and hepatic TAG concentration resulted (*r*^2^ = 0.2648; *r* = 0.5146; *P* = 0.0101) (Fig. 3a). However, the ratio of Kyn to Trp (KTR) (Fig. 3b) was decreased and no changes were observed for neopterin after TTP treatment (Table 1). Interestingly, KTR correlated to plasma antioxidant capacity (*r*^2^ = 0.2943; *r* = − 0.5425; *P* = 0.0200) (Fig. 3c), liver anti-inflammatory index (*r*^2^ = 0.6330; *r* = − 0.7956; *P* = 0.0020) (Fig. 3d), and liver atherogenicity index (*r*^2^ = 0.6068; *r* = − 0.7790; *P* = 0.0028) (Fig. 3e). Noteworthy, the hepatic gene expression of *Ido1* was significantly decreased by TTP administration whereas the liver mRNA level of *Tdo2* tended to increase, but these data were not statistically significant (Fig. 3f). This was followed by a reduced hepatic mRNA level of *Kynu* (Fig. 3f) and decreased plasma concentrations of AA and HAA (Table 1). The plasma metabolites KA and XA were also decreased by TTP treatment (Table 1) associated with decreased gene expression of *KatII*/*Aadat* (Fig. 3f) and unchanged expression of kynurenine-3-monooxygenase (*Kmo*). The strongest decrease in gene expression was observed with the key regulatory enzyme ACMSd (Fig. 3f). The enzyme activities were not measured, but these results could suggest that inhibition of the Trp-Kyn pathway is associated with the development of TTP-induced hepatic steatosis, without an increase in liver or plasma inflammation markers.

**Table 1:** Tryptophan and kynurenine pathway metabolites in plasma after 14 days of 0.9% TTP diet in C57BL/6 male mice (*n* = 6)

	Control	0.9% TTP	
	Mean ± SD	Mean ± SD	*P* -value
Trp (μM)	114.9 ± 5.92	144.4 ± 1.81	0.0147
Kyn (μM)	1.2 ± 0.02	1.3 ± 0.01	0.0346
Neopt (nM)	1.7 ± 0.10	2.1 ± 0.21	0.2182
AA (nM)	138.5 ± 7.84	83.5 ± 4.96	0.0069
HAA (nM)	21.8 ± 5.06	7.7 ± 0.24	0.0490
KA (nM)	150.9 ± 29.0	48.3 ± 5.19	0.0406
XA (nM)	147.3 ± 21.0	34.3 ± 0.48	0.0095
HK (nM)	86.3 ± 29.6	44.3 ± 14.0	0.1410
QA (nM)	291.0 ± 24.3	223.5 ± 48.8	0.3410
Nam (μM)	2.60 ± 0.37	4.49 ± 0.19	0.0160

*Abbreviations*: *AA* Anthranilic acid, *HAA* 3-hydroxyanthranilic acid, *HK* 3-hydroxykynurenine, *KA* Kynurenic acid, *Kyn* Kynurenine, *Neopt* Neopterin, *Nam* Nicotinamide, *QA* Quinolinic acid, *Trp* Tryptophan, *XA* Xanthurenic acid

### TTP-treatment modifies the hepatic NAD^+^ /NADH ratio and affects plasma metabolites of the Trp-Nam pathway

**Fig. 4:**
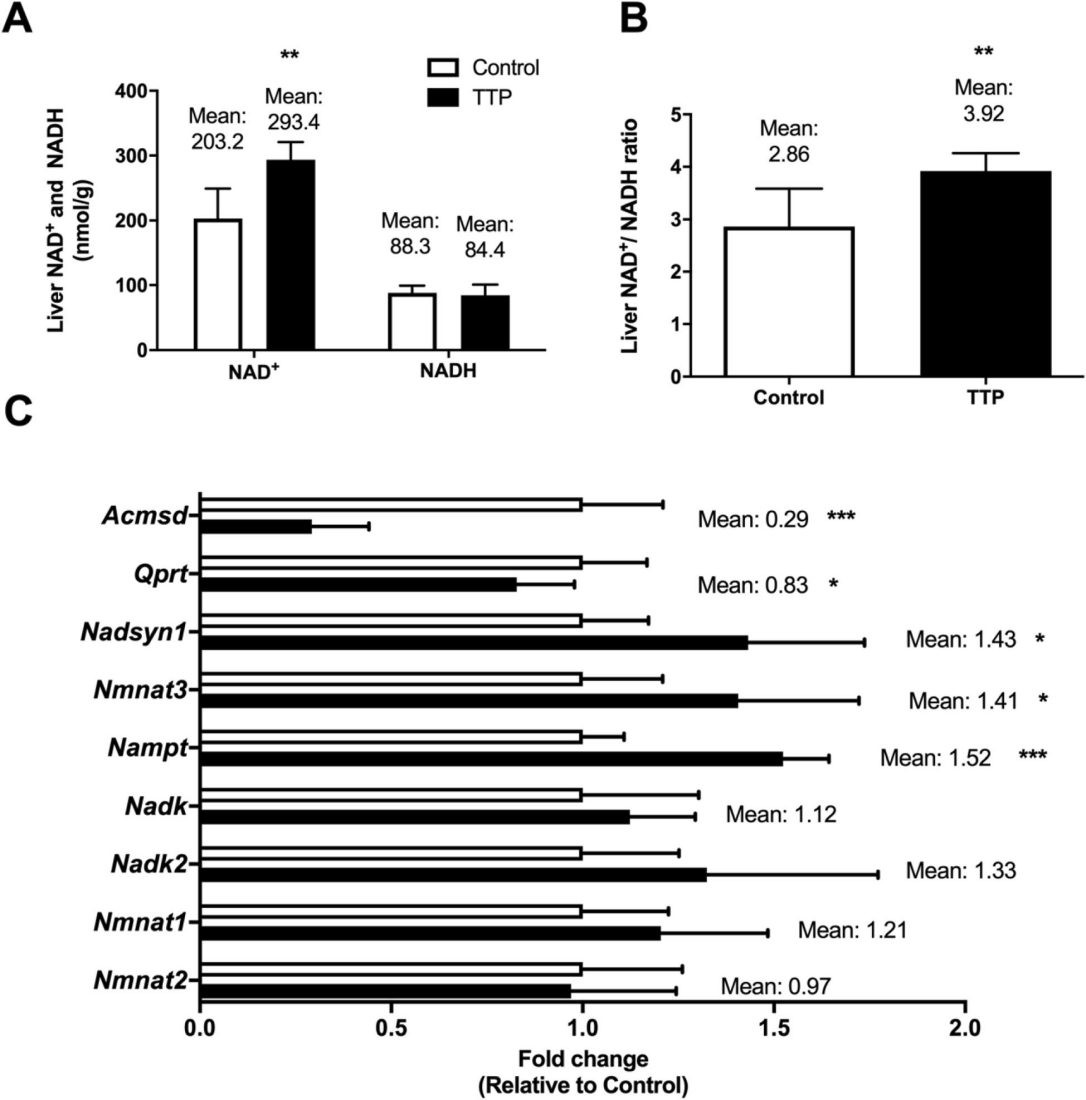
Liver NAD metabolites and gene regulation in TTP-treated rats. **a** Liver NAD^+^ and NADH levels and liver **b** NAD^+^ /NADH ratio. **c** Liver gene expression analysis. Data represented mean ± standard deviation. Statistical significance between control and TTP was shown as * *P* < 0.05; ** *P* < 0.02; *** *P* < 0.001

Interestingly, TTP increased the hepatic level of NAD^+^ accompanied by a significantly decreased hepatic mRNA level of *Acmsd* (Fig. 4a and c). However, the plasma concentration of QA remained constant (Table 1) and hepatic gene expression of *Qaprt* was reduced by dietary TTP (Fig. 4c). It was also tested whether the increase in NAD^+^ would be concomitant to changes in other NAD^+^ metabolites. Strikingly, the liver level of NADH remained unchanged by TTP treatment (Fig. 4a) whereas the plasma level of Nam was increased (Table 1). Thus. the hepatic NAD^+^ /NADH ratio was significantly increased during development of fatty liver (Fig. 4b). Analyses of gene expression included enzymes that could contribute to the increase in liver content of NAD^+^ and plasma Nam. Noteworthy, the hepatic mRNA levels of NAD synthetase, *Nmnat3* and *Nampt* were increased in TTP-treated animals, whereas the hepatic gene expression of NAD kinase, NAD kinase 2, and *Nmnat1* and − *2* were unaltered (Fig. 4c).

## Discussion

The results of the present study indicate that TTP-induced fatty liver was associated with changes in the kynurenine pathway in Trp metabolism. According to previous findings [15–17], TTP-induced hepatic steatosis-development is most likely due to decreased mitochondrial fatty acid oxidation, while the lipogenesis is unchanged [17]. In agreement with this in vitro fatty acid oxidation of palmitoyl-CoA was reduced in liver homogenates from TTP-treated mice. Interestingly, the activity of one of the main enzymes involved in peroxisomal fatty acid oxidation, ACOX, was increased but this did not prevent TAG accumulation in liver. Trp and its metabolites have been reported to induce hepatic steatosis [12], however conflicting reports in poultry suggest that Trp does not cause fatty liver [28, 29]. In the present study of TTP-induced fatty liver, the plasma Trp level increased and a positive correlation was observed between hepatic TAG concentration and plasma Trp (Fig. 3). However, we observed that the plasma level of all Kyn metabolites were decreased by TTP administration accompanied with decreased gene expression of *KatII*/*Aadat* and *Kynu*. These enzymes are localized to mitochondria or both mitochondria and cytosol [30, 31], and it is possible that the decreased level of Kyn metabolites are associated with decreased mitochondrial function and/or induced fatty liver.

In the present study it was found a strong downregulation of the enzyme ACMSDT [32], while the plasma level of QA and the hepatic gene expression of *Qaprt* were not changed. This was accompanied by an increase in the hepatic level of NAD^+^ without a concomitant change in the NADH level. It is not reasonable to assume that the decrease in plasma concentrations of kynurenine derivatives might indicate a faster conversion towards NAD. However, some authors have demonstrated that differences may exist between plasma and liver levels of intermediates in the Trp-Kyn pathway [33]. Liver Trp-Kyn metabolites have not been determined, but an important observation after TTP administration was a 2-fold increase of plasma Nam. Moreover, nicotinamide and NAD can be generated from nicotinic acid, and the diet contained 4% of AIN-93 vitamin mixture which constitute 120 mg/kg nicotinic acid. Whether the increased level of hepatic NAD^+^ after TTP administration come from the diet and not from Trp should be considered (Fig. 1). However, inhibition of mitochondrial FA oxidation could evoke NAD-dependent regulatory processes (for example, SIRT3 activation), cleaving NAD^+^ and thereby generating Nam. Thus, it is not clear why the liver would not recycle the Nam into NAD and cleave Trp instead. The gene expression of NAD synthetase, *Nampt*, and *Nmnat3* was increased in the TTP-treated animals. These data are consistent with the increased liver level of NAD^+^. Previous studies have reported that peroxisome proliferators elevated the liver NAD^+^ level and that this was due to an increase in NAD^+^ biosynthesis from Trp [34–36].

TTP is a weak PPARα ligand and increased the fatty acyl-CoA oxidase activity in liver (Fig. 2), and data suggest a relationship between the Trp-Nam pathway and PPARα pathways [33]. Whether the PPARα-dependent effect by TTP is responsible for the conversion of Trp to Nam should be considered. Interestingly, in peroxisome proliferator treated animals demonstrating a high hepatic fatty acid oxidation level, the NAD^+^/NADH ratio is decreased [36], whereas in the present model of fatty liver development with decreased mitochondrial fatty acid oxidation, the liver NAD^+^/NADH ratio was increased.

Increased liver inflammation is often related to hepatic steatosis [11] and the plasma KTR ratio increases during inflammation. Moreover, KTR is considered as a specific marker for the IDO activity and it is reported that plasma KTR is increased in patients with coronary disease [5]. In the present study, plasma KRT was decreased by TTP treatment. This was associated with decreased hepatic gene expression of *Ido*, but unchanged level of neopterin. Moreover, KTR was negatively correlated to the fatty acid anti-inflammatory index in plasma (data not shown) and liver, plasma antioxidant capacity and liver atherogenicity index (Fig. 3c-e). Thus, the fatty liver development by TTP was accompanied by an increased hepatic TAG content but was not associated with increased inflammation. In line with this, we have reported that TTP downregulated hepatic gene expression of IL-1β and TNFα but increased the mRNA level of cytosolic superoxide dismutase [17].

## Conclusion

In conclusion, we have demonstrated that TTP-induced mitochondrial dysfunction and hepatic steatosis can influence the Trp-Kyn- and Nam pathway and liver NAD^+-^level. This metabolic effect is not clear, but probably related to changes in the salvage pathway or from the diet, but not to an increased flux through the de novo NAD synthesis pathway resulting in an increased level of hepatic NAD^+^ without any increased systemic and local inflammation. Because fatty liver increased the liver ratio of NAD^+^/NADH while strong peroxisome proliferators decrease this ratio, the influence of mitochondrial proliferators on Nam-, NAD^+^ and NADH-metabolism should be evaluated. Further studies on the role of mitochondrial function will be of relevance in exploring novel therapies for the treatment of fatty liver, and in this respect the regulation of NAD synthesis will be of importance. Fatty acid analogues with opposite effects on mitochondrial function are available and these will be valuable tools in studies to clarify if improved mitochondrial function may antagonize the development of fatty liver. In the future fatty acid analogues or other PPAR ligands may be part of novel therapies for NAFLD.

## Data Availability

All data generated or analysed during this study are included in this published article, and raw data will be made available upon request.
